# A5 DEVELOPMENT OF A PRE-CLINICAL MOUSE MODEL TO INVESTIGATE ADVERSE FOOD REACTIONS IN INTESTINAL INFLAMMATION

**DOI:** 10.1093/jcag/gwad061.005

**Published:** 2024-02-14

**Authors:** B Barbosa da Luz, L Rondeau, R Dang, A Caminero

**Affiliations:** McMaster University, Hamilton, ON, Canada; McMaster University, Hamilton, ON, Canada; McMaster University, Hamilton, ON, Canada; McMaster University, Hamilton, ON, Canada

## Abstract

**Background:**

Patients with chronic intestinal conditions, including inflammatory bowel disease (IBD), experience diverse food-related adverse reactions. However,, the precise mechanisms of food intolerances in IBD remains unclear. Emerging evidence suggests that altered diet-microbiota interactions can contribute to the development of IBD. Previously we shown that intestinal microbiota plays an important role in the development of food intolerances. We hypothesize that microbial alterations in IBD patients facilitate adverse reactions to foods.

**Aims:**

To establish a preclinical mouse model to study adverse food reactions in intestinal inflammation.

**Methods:**

Eight-week-old C57BL/6 mice were treated with 3% dextran sodium sulfate (DSS) for 5 days, then divided into three groups. The first group was sensitized to dairy protein (3% casein and 3% whey), using 4 oral administrations of cholera toxin over 2 weeks, without any further diet intervention. The second group was also sensitized to casein and whey, then after 1 week of recovery, the mice were subsequently place on a chow containing casein and whey. Finally, the third group was subjected to diet intervention without sensitization. The same experiment was repeated in a different subset of C57BL/6 mice which included a second cycle of DSS (1.5%) during the dietary interventions. Markers of intestinal inflammation (disease activity index (DAI), histological damage, cell infiltration and pro-inflammatory genes expression (NanoString)) and intestinal microbiota were analysed after 1 week of the diet intervention.

**Results:**

Mice previously sensitized on the dairy diet exhibited an enhanced activation of immune cells after intestinal inflammation. While the histological damage score did not reveal significant differences among experimental groups, the sensitized group under dairy intervention exhibited an elevated level of immune cell infiltration, increasing the polymorphonuclear, CD3^+^ and mast cells. Gene expression also revealed that dairy induced the expression of inflammatory genes, such as *C6*, *Arg1*, *Tnf*, *Il6*, *Cxcl9* and *Cxcl10*. When animals are subjected to a second DSS-cycle, dairy worsen colitis in mice previously sensitized, as shown by higher DAI compared to group with control diet and dairy diet without sensitization.

**Conclusions:**

In the context of intestinal inflammation, dairy protein leads to immune activation characterized by an increase in polymorphonuclear cells, mast cells and lymphocytes, as well as worsening colitis. Future studies using this model will provide valuable insights into the role of intestinal microbiota in food intolerances associated with intestinal inflammation.

**Funding Agencies:**

CCC

